# A Case of General Anæsthesia

**Published:** 1858-10

**Authors:** 


### *A Case of General Anæsthesia.* By Dr. KLAATSCH.

CASE. Dr. Klaatsch relates the case of a widow, æt. 58, who applied to him on account of severe pains in the extremities, and a powerless state of the upper ones, which prevented her from grasping any object firmly. She also complained of an unrelievable sense of hunger. In other respects, with the exception of occasional headaches, she was quite well. She had had nine children, still menstruated moderately, and exhibited no symptom of hysteria. A charwoman by occupation, she attributed her present symptoms to chills. An examination exhibited no appearances of paralysis, but the sense of feeling was lost over the entire skin and orifices of the mucous membranes, and could not be excited by pricking with needles. No unpleasant feeling was excited by irritating the nares, the conjunctivæ, or the mucous membrane of the mouth, and the fumes of ammonia produced no effect upon the respiratory organs. Boiling water and a prolonged application of the electrical pencil alone induced some feeling on the surface. The sense of contact was retained, and she was enabled to exactly indicate the spots at which she had been pricked. The power of distinguishing between differences of temperature was abolished, but the muscular sense remained, as she was conscious of the position of her limbs, and could determine the weight and size of a body by grasping it. The senses of taste and smell were lost, and both hearing and vision were somewhat defective, although she had not before observed diminution in their power. The reflex excitability was very slight, the rapid application of hot sponges exciting but slight movements; tickling the fauces did not induce any disposition to vomit.

After the patient had remained some time under observation, and her condition was found to be stationary, the Russian vapor-baths were tried, but without any avail. The electrical pencil was next applied to the two forearms and the left side of the face; and after three *séances*, not only was feeling restored to these parts, but it was recovered also by the remainder of the surface and the mucous membranes; taste and smell were restored, and the sense of unappeasable hunger disappeared.

Most of the analogous cases on record have been observed in hysterical subjects; but in this one no symptoms of hysteria whatever were present.—*Deutsche Klinik*, 1856, and *Med. Times and Gaz.*, from *Ranking's Abstract of Med. Sciences.*

